# An examination of the psychometric properties of the Patient Health Questionnaire-9 (PHQ-9) in a Multiracial/ethnic population in the United States

**DOI:** 10.3389/fpsyt.2023.1290736

**Published:** 2024-01-16

**Authors:** Jaimie Shaff, Geoffrey Kahn, Holly C. Wilcox

**Affiliations:** ^1^Johns Hopkins Bloomberg School of Public Health, Baltimore, MD, United States; ^2^Henry Ford Health, Detroit, MI, United States

**Keywords:** depression, patient health questionnaire, factor analysis, statistical, psychometrics, racial groups, United States

## Abstract

Depression and suicide are significant public health issues. The Patient Health Questionnaire-9 (PHQ-9) is commonly used to assess for symptoms of depression, but its psychometric properties within Multiracial/ethnic populations remains uncertain. In a study involving 1,012 English-speaking Multiracial/ethnic participants from the United States (US), the PHQ-9 showed strong internal consistency (*α* = 0.93) and supported a one-factor structure. No measurement variance was observed between Non-White and White/Non-White Multiracial/ethic subgroups. PHQ-2, with a cutoff of ≥3, identified fewer depression cases than PHQ-9 (32% vs. 40%), with sensitivities of 75–99% and specificities of 74–96%; a cutoff of ≥2 missed fewer cases. Item performance of the ninth PHQ-9 question, addressing thoughts of death or self-harm, varied across generations with younger generations more likely to endorse thoughts of death or self-harm at any level of symptom severity. The findings suggest the PHQ-9 demonstrated adequate reliability within a population of Multiracial/ethnic adults in the US; however, the use of the 9th item of the PHQ-9 may not be adequate for identifying individuals at risk for suicidal thoughts and/or behaviors, particularly for older Multiracial/ethnic adults. The lower sensitivity of the PHQ-2 with a ≥ 3 cutoff suggests a cutoff of ≥2 may be preferable to miss fewer cases of depression.

## 1 Introduction

Depression and suicide are critical public health issues that impact individuals, families, and society at large. During and following the COVID-19 pandemic, the United States (US) experienced an increase in the prevalence of depressive symptoms for all racial and ethnic groups and an increase in suicide rates among adolescents and American Indian, Black, and Latino adults (1). As of June 2023, the US Preventive Services Task Force (USPSTF) recommends screening all adolescents and adults for depression (2).

The Patient Health Questionnaire-9 (PHQ-9) is an instrument used to assess for symptoms of depression consistent with Diagnostic and Statistical Manual of Mental Disorders, Fifth Edition (DSM-5) criteria (3). The PHQ-9 has demonstrated robust psychometric properties in culturally, linguistically, and geographically diverse samples of adults, contributing to its widespread usage (4). Two questions from the PHQ-9, known as the PHQ-2, are often used as a brief measure and is currently used by the CDC in their routine Household Pulse Survey (1). The ninth item of the PHQ-9 asks about “thoughts that you would be better off dead, or of hurting yourself.” The ninth item of the PHQ-9 is used in primary care and other settings to identify individuals at risk for suicidal thoughts and behaviors; however, studies have indicated this to be an insufficient assessment tool for suicide risk (5–8).

In the US, Multiracial/ethnic populations are a rapidly growing demographic group that has been historically underrepresented in public health surveillance, research and practice (9, 10). Population growth appears to be increasing across generations: the 2021 American Community Survey estimates that 7.7% of Baby Boomers (born in or before 1964), 11.7% of Generation X (1965–1980), 12.9% of Millennials (1981–1996), and 16.6% of Generation Z (1997 or later) identify with two or more racial groups (11). Emerging research in the past decade suggests Multiracial/ethnic populations in the United States may have the highest prevalence of many mental health conditions, including depression and suicide (1, 12, 13). While studies have provided evidence to suggest the effectiveness of the PHQ-9 as a depression screener within White, Black, African American, Asian, Chinese American, Mexican American, and Latino populations in the US, partial measurement invariance was found for the one-factor model in a population of American Indian/Alaska Native adults, supporting efforts to continue examining the appropriateness of this tool within diverse populations (14–17). To the authors’ knowledge, studies have yet to establish the psychometric properties of the PHQ-9 within Multiracial/ethnic populations in the US.

This study aims to investigate the psychometric properties of the PHQ-9 within a sample of Multiracial/ethnic adults in the United States. This study explored the following research questions: 1) Does the PHQ-9 have adequate psychometric performance in a Multiracial/ethnic adult population in the US? 2) Is psychometric performance comparable across generations and between White and non-White Multiracial/ethnic people? 3) How does the PHQ-2 compare with the PHQ-9 at identifying clinically meaningful depression in this population?.

## 2 Methods

A nonprobability-based convenience sample of English-speaking adults living in or from the United States that identify as multiracial and/or multiethnic and selected at least two distinct categories for racial/ethnic identity (White, Black or African American, American Indian or Alaska Native (AI/AN), Asian, Native Hawaiian or Pacific Islander, Middle Eastern or North African, Other) was obtained through an online anonymous survey collected from October–December 2022. Respondents were recruited from multiple market research panels facilitated by Qualtrics, which aims to mirror census representation, with compensation up to $9.50 (18). The Johns Hopkins Bloomberg School of Public Health Institutional Review Board (IRB) approved this study and informed consent was obtained.

### 2.1 Measures

Depressive symptoms and severity were assessed using the 9- item Patient Health Questionnaire (PHQ-9), a validated tool based on the DSM-5 criteria (3). On each item of the PHQ-9, participants were asked “Over the past 2 weeks, how often have you been bothered by any of the following?” and provided responses on a 4-point Likert scale (0 = Not At All, 1: Several Days, 2: More than Half the Days, 3: Nearly Every Day). The PHQ-2 consists of the first two questions of the PHQ-9, “Little interest or pleasure in doing things,” and “Feeling down, depressed, or hopeless.” The survey collected demographic data on racial and ethnic identity, gender identity, sexual orientation, age, place of birth, educational attainment, and household income level. Participants were split into four age groups based on birth year: Gen Z was defined as born in 1997 or later, Millennial was born 1981–1996, Gen X was born 1965–1980, and Baby Boomers were born 1964 and earlier. To explore within-group differences, participants were split into two race groups: those who endorsed White as one of their racial/ethnic identities and those who did not.

### 2.2 Statistical analysis

The internal consistency of the PHQ-9 was measured using Cronbach’s alpha and McDonald’s omega. An exploratory factor analysis was conducted and scree plot examined to identify the number of latent factors. Measurement invariance was tested across two variables: age and race, defined above. In both cases, measurement invariance was tested by fitting a series of confirmatory factor analyses. In one model, factor loadings were constrained to be equal between groups and in another they were freely estimated. A chi-squared difference test was then used to compare models. In the event of a significant test, score tests were used to identify which questions differed between which groups. If the test was non-significant, the process was repeated constraining model intercepts and finally residuals (i.e., testing metric, scalar, and then strict invariance). If any of the tests were significant, further invariance testing was not done. The weighted least-squares estimator was used with robust standard errors calculated using the full weight matrix.

The PHQ-2 was compared to the PHQ-9 as a screening tool for depression. Using a threshold of ≥3 for the PHQ-2 and the PHQ-9 as the gold standard, the sensitivity, specificity, positive predictive value (PPV), and negative predictive value (NPV) were calculated for moderate, moderately severe, and severe depression (PHQ-9 ≥ 10, 15, and 20, respectively). Performance measures were also calculated for PHQ-2 thresholds of ≥2, ≥3, and ≥ 4. Based on observed measurement variance of the PHQ-9 between generations, a *post-hoc* analysis was also conducted using a threshold of ≥3 stratified by generation.

All analyses were conducted using R Statistical Software version 4.2 (19) and the packages psych, lavaan, and semTools (20–22).

## 3 Results

### 3.1 Sample characteristics

The sample (*N = 1,012*) was majority female (67.5%, *n = 683*) and straight (80.1%, *n = 798*). More than half had attained less than a college degree (62.3%, *n = 627*), and about half reported a household income less than $60,000 (57.4%, *n = 552*). The mean birth year of the sample was 1981 (SD = 14.4). Almost half (43%, *n = 435*) of respondents were born between the years 1981–1996 and classified as Millennials; 27.4% (*n = 277*) between 1965 and 1980, classified as Gen-X; 15% *(n = 152*) after 1997, classified as Gen-Z; and 14% between 1946 and 1964, classified as Baby Boomers. Less than 1% of the sample were born before 1946. Over half of respondents (55%, *n = 557*) reported identifying as part-White; 48.2% (*n = 488*), Black or African American; 48.1% (*n = 487*), Hispanic or Latino; 16.3% (*n = 165*), Asian; 29.4% (*n = 298*), American Indian or Alaska Native; 8.5% (*n = 86*), Native Hawaiian or Pacific Islander; 8.1% (*n = 82*), Middle Eastern or North African; 8.9% (*n = 90*) identified with a racial or ethnic group not listed in these broad categories.

### 3.2 PHQ-9 psychometric properties

**Figure 1:**
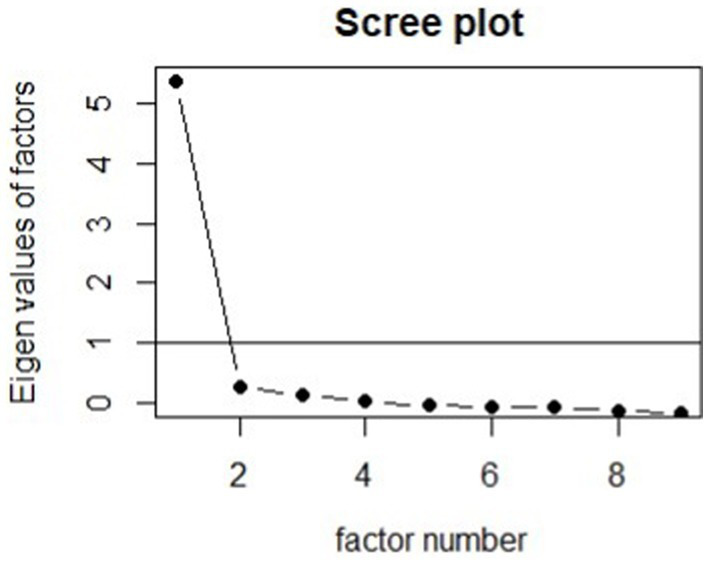
Scree plot for the PHQ-9. In this Multiracial/ethnic sample, the PHQ-9 appears to measure a single latent factor.

The Cronbach’s alpha for the PHQ-9 in this sample was 0.93 (95% CI, 0.92–0.94) and McDonald’s omega was also 0.93 (95% CI, 0.91–0.94). The factor analysis confirmed a one factor solution was the best supported; the scree plot is shown in Figure 1. The first factor had an eigenvalue of 5.76 and explained 59.7% of the variance. The factor loadings and uniqueness are shown in Table 1. When loadings were estimated separately by generation, the unconstrained model fit the data significantly better (chi-squared difference 55.226 on 24 degrees of freedom, *p* = 0.0003). Score tests revealed that the loading of the 9th item differed significantly between all generations. Specifically, it decreased with increasing age, so the loadings were 0.900, 0.690, 0.471, and 0.361 for Gen Z, Millennials, Gen X, and Baby Boomers, respectively. When loadings, intercepts, and residuals were estimated separately by race group, the unconstrained model did not fit the data significantly better (chi-squared difference 30.4 on 25 degrees of freedom, *p* = 0.2097), suggesting strict measurement invariance between race groups. The fit statistics for the final measurement model were: robust RMSEA 0.041, CFI 0.994, TLI 0.995, and SRMR 0.047.

**Table 1:** Factor loading and uniqueness for items in the PHQ-9.

Item	Loading	Uniqueness
Question 1: Little interest or pleasure in doing things	0.830	0.311
Question 2: Feeling down, depressed, or hopeless	0.856	0.267
Question 3: Trouble falling or staying asleep, or sleeping too much	0.740	0.452
Question 4: Feeling tired or having little energy	0.782	0.388
Question 5: Poor appetite or overeating	0.793	0.371
Question 6: Feeling bad about yourself — or that you are a failure or have let yourself or your family down	0.817	0.332
Question 7: Trouble concentrating on things, such as reading the newspaper or watching television	0.804	0.354
Question 8: Moving or speaking so slowly that other people could have noticed? Or the opposite — being so fidgety or restless that you have been moving around a lot more than usual	0.669	0.552
Question 9: Thoughts that you would be better off dead or of hurting yourself in some way	0.633	0.600
Question 9 loading estimated separately for each generation		
Gen Z	0.900	–
Millennial	0.690	–
Gen X	0.471	–
Baby Boomer	0.361	–

### 3.3 PHQ-2 performance

The PHQ-2 identified significantly fewer people overall as depressed compared to the PHQ-9 (32% vs. 40%, *p* < 0.001). Using a PHQ-2 score threshold of 3, the sensitivity for detecting PHQ-9 mild, moderate, and severe depression was 75, 95, and 99%, respectively. The specificity was 96, 85, and 74%, respectively. A threshold of 3 performed better overall than thresholds of 2 or 4; full details are shown in Table 2. PHQ-2 performance was generally comparable between generations. Using a threshold of 3, the sensitivity for mild depression varied 72–76% between generations. The specificity was 90% for Gen Z and varied 96–97% for older generations.

**Table 2:** Comparing PHQ-2 score thresholds for detecting depression.

PHQ-2 threshold	PHQ-9 depression	Sensitivity	Specificity	PPV	NPV
2	Moderate	94%	70%	68%	95%
	Moderately Severe	99%	57%	39%	99%
	Severe	100%	49%	17%	100%
3	Moderate	73%	96%	92%	85%
	Moderately Severe	95%	85%	64%	98%
	Severe	99%	74%	28%	99%
4	Moderate	55%	99%	97%	77%
	Moderately Severe	78%	93%	75%	94%
	Severe	94%	85%	38%	99%

## 4 Discussion

This study begins to fill a gap in the literature on the performance of mental health instruments within Multiracial/ethnic populations, a population underrepresented in public health research. To the author’s knowledge, psychometric assessments of the PHQ-9 for this population have not been published. This study provides evidence to suggest the PHQ-9 demonstrates high reliability with a one-factor solution within Multiracial/ethnic adult populations in the US, suggests the PHQ-9 is an appropriate depression screening instrument for Multiracial/ethnic adult populations in the US, and joins prior studies demonstrating the utility of the PHQ-9 within some racially and ethnically diverse US populations (15, 17).

This study has several limitations. As a cross-sectional study conducted primarily among English-speaking Multiracial/ethnic adults with internet access and recruited via paid research panels, results may not generalize to all Multiracial/multiethnic people in the US. We included Hispanic and Latino Multiracial/ethnic people in our study population, so our results may have limited comparability to data that exclude Hispanic and Latino people from Multiracial categories. As few studies have been conducted among Multiracial/ethnic adult populations, there is no standardized or proposed approach for assessing within-group racial/ethnic differences, limiting the ability to analyze detailed racial/ethnic differences and comparability of results to future studies within Multiracial/ethnic populations (9). Finally, as the study lacks a formal clinical diagnostic element, we were unable to test the validity of the PHQ-9 within this sample.

### 4.1 Implications for Research and Practice

In an analysis across generations of adults, the study found variable measurement with respect to generation, with a particularly salient finding regarding the ninth question of the PHQ-9 which assesses for thoughts of death or self-harm. Specifically, the study found that the 9th item had progressively lower correlation with the underlying latent factor (i.e., depression) among older generations. A *post hoc* analysis suggested that older adults were less likely to report thoughts of death or self harm even when reporting high levels of other depressive symptoms. Additionally, Gen Z were more likely to report thoughts of death or self harm even at lower levels of other depressive symptoms than other generations. This finding aligns with a 2018 report by the American Psychological Association that found Gen-Z more open about reporting concerns related to mental health, and suggests the utility of a slightly tailored approach when using the PHQ-9 with different generations of Multiracial/ethnic adults (23). The use of the 9^th^ item of the PHQ-9 may not be adequate for identifying individuals at risk for suicidal thoughts and/or behaviors, particularly for older Multiracial/ethnic adults, which may present an ethical challenge. The use of an additional tool to identify those at risk for suicide is recommended, as feasible. There is a need to conduct further research within diverse populations to explore for similar differences across age groups and explore the causes underlying this differential reporting of thoughts of death or self-harm to adequately inform public health research, surveillance, and clinical interventions.

This study found no evidence of measurement variance between Non-White and White/Non-White sub-populations. This study supports findings from a 2010 systematic review by Kroenke et al. that detailed the reliability of the PHQ-9 across populations and sample types, but was unable to support findings from prior research among and racially, ethnically, and linguistically diverse populations that preferred a two-factor solution (14, 17, 24). However, robust analyses of the psychometric properties of the tool by various Multiracial/ethnic constructs were not possible due to the limited sample of this exploratory study. Given the exploratory findings by generation and questions about the appropriateness of this tool within diverse samples, future research including a diagnostic element that can also examine results by different Multiracial/ethnic constructs (ie. White & Asian, White & Black, White & AI/AN, Black & Asian, Black and AI/AN, Black & White & Asian, etc.) are warranted. Future research conducted among international samples should also consider their local Multiracial/ethnic populations, and ensure psychometric assessments include these populations.

As the PHQ-2 is a widely adopted brief measure within the field of public health, this study explored the reliability and validity of the PHQ-2 at different cutoffs, using the PHQ-9 as the gold standard. Findings on the PHQ-2 align with prior evidence suggesting the adequacy of the commonly used cutoff of “3” to identify those endorsing symptoms of moderately severe or severe depression, with the recommendation of using the cutoff of “2” to miss fewer cases (25). These findings support a 2019 systematic review and meta-analysis and a 2016 diagnostic meta analysis that suggest reducing the cutoff to “2” to capture more potential cases, with the trade-off being the identification of a higher proportion of false positives (25, 26).

This study makes an important contribution to the literature by finding high internal consistency and support for measurement invariance of the one-factor PHQ-9 model within a sample of Multiracial/ethnic adults in the US. Additionally, this study provides critical information that questions the use of the 9th question alone as a suicide screener, particularly in mixed age populations. As many public health workers and health care providers seek to streamline screening and assessment processes, this study provides evidence to support the use of the PHQ-2 with Multiracial/ethnic adult populations in the US with a recommended cutoff of 2 to capture the most potential cases. The PHQ is one of the most commonly used depression screening tools and Multiracial/multiethnic people are a diverse and growing population presently underrepresented in psychometric studies. It is important to have evidence that this common and important tool likely functions similarly for Multiracial/multiethnic people as for other clinical populations.

## Data availability statement

The participants of this study did not give written consent for their data to be shared publicly. With permission by the Johns Hopkins University IRB, an anonymized dataset from the quantitative study can be made available upon written request to the corresponding author.

## Ethics statement

The studies involving humans were approved by Johns Hopkins University Institutional Review Board. The studies were conducted in accordance with the local legislation and institutional requirements. The participants provided their non-identifying written informed consent to participate in this study.

## Author contributions

JS: Conceptualization, Data curation, Formal analysis, Funding acquisition, Investigation, Methodology, Project administration, Writing – original draft, Writing – review & editing. GK: Data curation, Formal analysis, Methodology, Writing – original draft, Writing – review & editing. HW: Conceptualization, Funding acquisition, Methodology, Project administration, Supervision, Writing – review & editing.
